# Agreement of Gonial Angle Measurements Between Cone-Beam Computed Tomography (CBCT)-Reconstructed Panoramic Radiographs and Conventional Panoramic Radiographs: A Cross-Sectional Study

**DOI:** 10.7759/cureus.113513

**Published:** 2026-07-28

**Authors:** Apurba Gupta, Harshavardhan Kidiyoor, Roopak D Naik

**Affiliations:** 1 Orthodontics and Dentofacial Orthopaedics, Sri Dharmasthala Manjunatheshwara (SDM) College of Dental Sciences, Shri Dharmasthala Manjunatheswara University, Dharwad, IND

**Keywords:** cone beam computed tomography, gonial angle, lateral cephalogram, normodivergent, orthopantomogram, three-dimensional imaging

## Abstract

Introduction

The gonial angle is an important cephalometric parameter used to assess mandibular morphology and vertical facial growth. Conventional lateral cephalograms (LCs) and orthopantomograms (OPGs) are routinely used for its evaluation; however, image distortion and superimposition may affect measurement accuracy. Cone-beam computed tomography (CBCT) enables 3D visualization and can generate reconstructed 2D cephalometric and panoramic images that may provide comparable measurements without requiring additional radiographic exposure when a CBCT scan is already available.

Objective

To evaluate the agreement between gonial angle measurements obtained from conventional LCs and OPGs and those derived from CBCT-reconstructed cephalograms and panoramic images in normodivergent individuals.

Methods

This cross-sectional study included 24 normodivergent orthodontic patients. Gonial angles were measured on conventional LCs, conventional OPGs, CBCT-reconstructed cephalograms, and CBCT-generated panoramic images using standardized digital imaging software. Mean measurements were compared using paired statistical analysis. Agreement between the imaging modalities was assessed using Bland-Altman analysis, while intra- and inter-examiner reliability were evaluated using intraclass correlation coefficients (ICCs) and weighted kappa statistics.

Results

No statistically significant differences were observed between gonial angle measurements obtained from conventional radiographs and those obtained from CBCT-reconstructed images (p > 0.05). Bland-Altman analysis demonstrated minimal systematic bias and acceptable limits of agreement between the imaging modalities. Both conventional and CBCT-derived measurements exhibited excellent intra- and inter-examiner reliability, supporting the reproducibility of the measurement techniques.

Conclusions

CBCT-reconstructed cephalograms and panoramic images demonstrated excellent agreement with conventional LCs and OPGs for gonial angle assessment in normodivergent individuals. When a CBCT scan has already been acquired for clinical indications, reconstructed images may provide reliable gonial angle measurements without the need for additional conventional radiographs, thereby reducing unnecessary radiation exposure.

## Introduction

The gonial angle is an important factor in diagnosis and treatment planning in orthodontics. It plays a pivotal role in evaluating mandibular rotation [1] and in assessing the vertical growth pattern, which may influence orthodontic diagnosis and treatment planning [2]. Along with mandibular shape and mental form, the gonial angle may be used as a landmark for forensic identification, particularly in cases involving mass disasters, exhumed skeletal remains, homicidal dismemberment, and unidentified individuals [3].

Any increase or decrease in the gonial angle beyond normal values may indicate disharmony of the craniofacial skeleton. Individuals exhibiting downward and backward rotation of the mandible are identified as having a “high-angle” pattern, with a large gonial angle, whereas those exhibiting upward and forward rotation of the mandible are identified as having a “low-angle” pattern, with a smaller gonial angle [4].

This angle is relevant in orthodontic research for understanding changes during the developmental period and is associated with the function and shape of the muscles of mastication. Strong activity of the masseter and anterior temporalis muscles is typically characteristic of a small gonial angle [5].

Traditionally, lateral cephalograms (LCs) have been used to measure the gonial angle. However, conventional 2D lateral cephalometric analysis has several inherent limitations when evaluating 3D craniofacial structures. This is due to the projection of complex 3D structures onto a 2D sagittal plane, resulting in a flattened and distorted image [6]. Additionally, variations in head position and imaging geometry may increase magnification and the superimposition of bilateral osseous structures, such as those forming the gonial angle. Such distortions can complicate landmark identification and measurement accuracy, particularly for structures located away from the midsagittal plane [7]. The use of orthopantomograms (OPGs) has been suggested so that the right and left gonial angles can be measured individually and accurately, without bilateral superimposition. However, studies comparing the two modalities have reported conflicting findings [8-11]. Most studies using 2D imaging techniques have considered the gonial angle as the average measurement of a bilateral structure superimposed on tracings.

Multislice computed tomography (MSCT) has enabled accurate 3D visualization of craniofacial anatomy, reducing the problem of overlapping structures and improving the assessment of skeletal relationships [12]. However, its routine use in orthodontics has been limited by substantially higher radiation exposure than that associated with conventional 2D cephalometric imaging. The subsequent development of cone-beam computed tomography (CBCT) provided high-resolution 3D imaging with a comparatively lower radiation dose than MSCT, rendering it useful in craniofacial analysis [13].

Sophisticated 3D imaging techniques such as CBCT may be an alternative to conventional 2D radiographic imaging, as they allow the acquisition of anatomically accurate images of the jaws at a fraction of the cost and radiation exposure associated with traditional computed tomography imaging [14-16]. Because standard population norms have not been available for CBCT volumes, patients may be subjected to the additional radiation exposure associated with traditional LCs and panoramic radiographs to acquire accurate data [17]. Thus, the possibility that CBCT imaging could supplement and/or replace conventional radiographs in diagnosis and treatment planning has not been fully explored.

The aim of this study was to evaluate the agreement between gonial angle measurements obtained from conventional LCs and OPGs and those obtained from CBCT-reconstructed cephalograms and panoramic images in normodivergent individuals.

## Materials and methods

Study design and setting

The sampling frame for this study consisted of official clinic appointment logs for patients undergoing orthodontic treatment at the Department of Orthodontics, SDM College of Dental Sciences, Sattur, Dharwad, Karnataka. Individuals who met the inclusion criteria were selected to participate in this cross-sectional study. Informed consent to participate was obtained from all participants. The study was conducted over three years, from 2020 to 2023. Ethical approval was obtained from the Institutional Review Board of Shri Dharmasthala Manjunatheshwara University, SDM College of Dental Sciences and Hospital, Dharwad, Karnataka, India (approval no. SDMCDS IEC. No. 2021/P/OR/70).

Sample size estimation

The sample size was determined in consultation with a statistician using a paired-comparison study design. Assuming a two-sided significance level (α) of 0.05, statistical power of 95%, and an effect size of 0.7564 derived from the study by Park CS et al. [18], the minimum required sample size was calculated to be 24 participants.

Study sample

The inclusion criteria included individuals aged 15-30 years who reported to the Department of Orthodontics for treatment, exhibited a skeletal Class I relationship, and had no gross asymmetry, more than minimal tooth rotation, or history of craniofacial anomalies. In addition, dentate individuals with minimal tooth loss due to caries or extractions and a mandibular plane angle of 21.9° ± 3.2° according to Downs analysis were selected for inclusion in the study. The age range of 15-30 years was selected to ensure that major pubertal craniofacial growth was complete, thereby preventing confounding due to changes in skeletal growth from affecting the outcome.

Individuals displaying mandibular skeletal asymmetry or having a history of trauma, mandibular or facial surgery, or any medical syndrome affecting the facial skeleton were excluded from the study. Additionally, individuals with temporomandibular joint disorders or condylar abnormalities were excluded.

Data collection and image reconstruction protocol

The LCs and panoramic images of the selected individuals were obtained using the Carestream Dental CS 9600 imaging system (Atlanta, GA, USA; version 8.018) and a complementary metal-oxide-semiconductor sensor [19]. The imaging parameters were 80 kV, 10.0 mA, and an exposure time of 10 seconds for the LCs and 73 kV, 8.0 mA, and an exposure time of 12.3 seconds for the panoramic images, respectively.

Clinically indicated CBCT scans were obtained from orthodontic patients for various purposes, including the placement of temporary anchorage devices (TADs) and the assessment of supernumerary teeth. CBCT images of the mandible were obtained using the Carestream Dental CS 9600 imaging system (Atlanta, GA, USA; version 8.018), a complementary metal-oxide-semiconductor sensor, and dental volumetric reconstruction. The imaging parameters were 120 kV, 5 mA, an exposure time of 12 seconds, and a field of view of 16 × 10 cm, which is often used for orthodontic diagnosis in conjunction with interdisciplinary treatment needs. The scans were obtained using the Mandibular CBCT Scan tool in the Carestream software. All scans were obtained by the same technician using the same device, with the patients positioned in the natural head position. The principal investigator and a senior orthodontist, both well versed in cephalometric landmark assessment, carried out the measurements as described. The study followed a single-blind design.

The obtained CBCT, lateral cephalometric, and panoramic images were opened and viewed using CS 3D Imaging software, version 3.8. The software allowed the measurement of specific linear and angular relationships between anatomical points, including the gonial angle. On the radiographs, lines tangent to the lower border of the mandible (mandibular plane) and the posterior border of the ramus and condyle (ramal plane) were visualized and used as references for marking the following points on the digital images: articulare, gonion, and menton.

**Figure 1:**
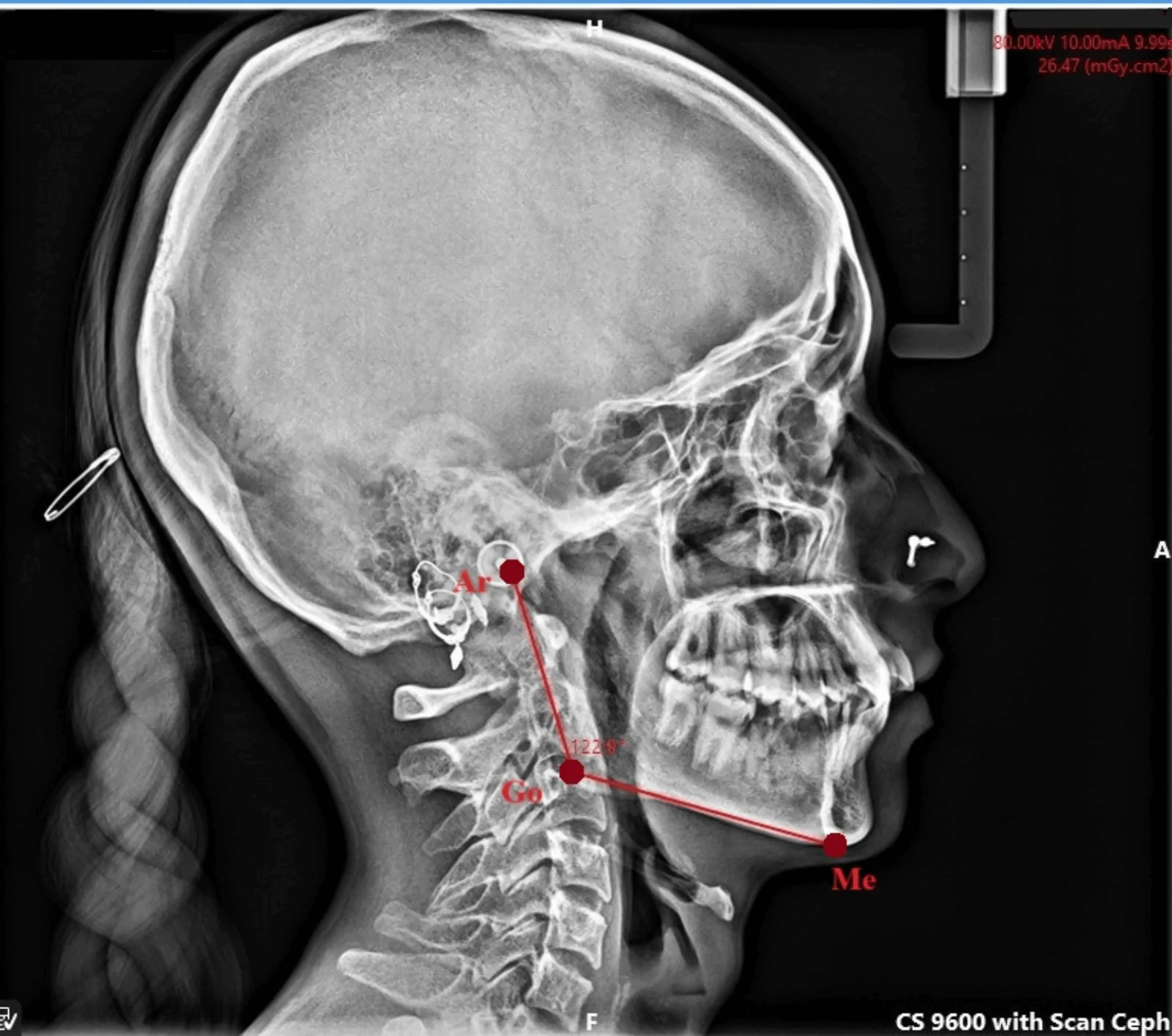
Lateral cephalographic image showing the gonial angle (Ar-Go-Me; red), measured between a line tangent to the posterior border of the mandibular ramus and a line tangent to the inferior border of the mandible. The measurement was obtained using the angle measurement tool in Carestream 9600 imaging software, version 8.018. Ar: Articulare; Go: Gonion; Me: Menton.

The points articulare (the point at the junction of the posterior border of the ramus and the inferior border of the cranial base (occipital bone)), gonion (the point on the curvature of the angle of the mandible, located by bisecting the angle formed by lines tangent to the posterior ramus and the inferior border of the mandible), and menton (the lowest point on the symphyseal shadow of the mandible observed on a LC) were marked using the cursor after selecting the Angle Measuring Tool provided in the left-hand panel of the software (Figure 1).

**Figure 2:**
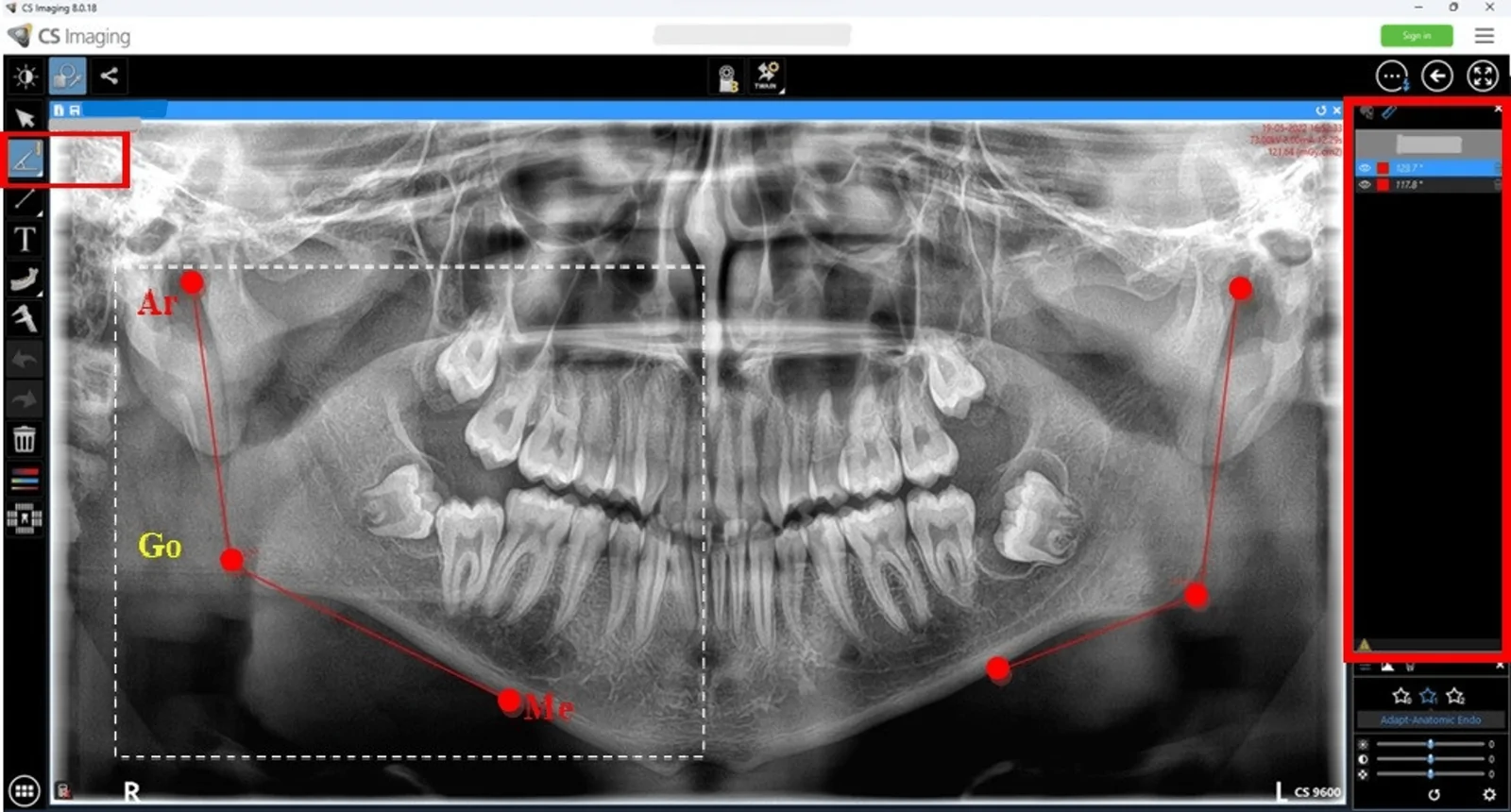
Orthopantomogram showing the gonial angle (Ar-Go-Me; red), measured between a line tangent to the posterior border of the mandibular ramus and a line tangent to the inferior border of the mandible. The measurement was obtained using the angle measurement tool in Carestream 9600 imaging software, version 8.018. Ar: Articulare; Go: Gonion; Me: Menton; OPG: Orthopantomogram.

Once the three points had been marked, the angle and its numerical value were automatically displayed diagrammatically in red on the image and numerically in the display panel on the right side of the window. This was performed on the LC and on the right and left sides of each panoramic image. The evaluator repeated the measurements multiple times to minimize measurement inaccuracies (Figure 2).

For each CBCT image, the Curved Slicing option was selected to view the image. This was followed by 2D reconstruction of the CBCT data by tracing the dental arch using the reconstruction tool.

**Figure 3:**
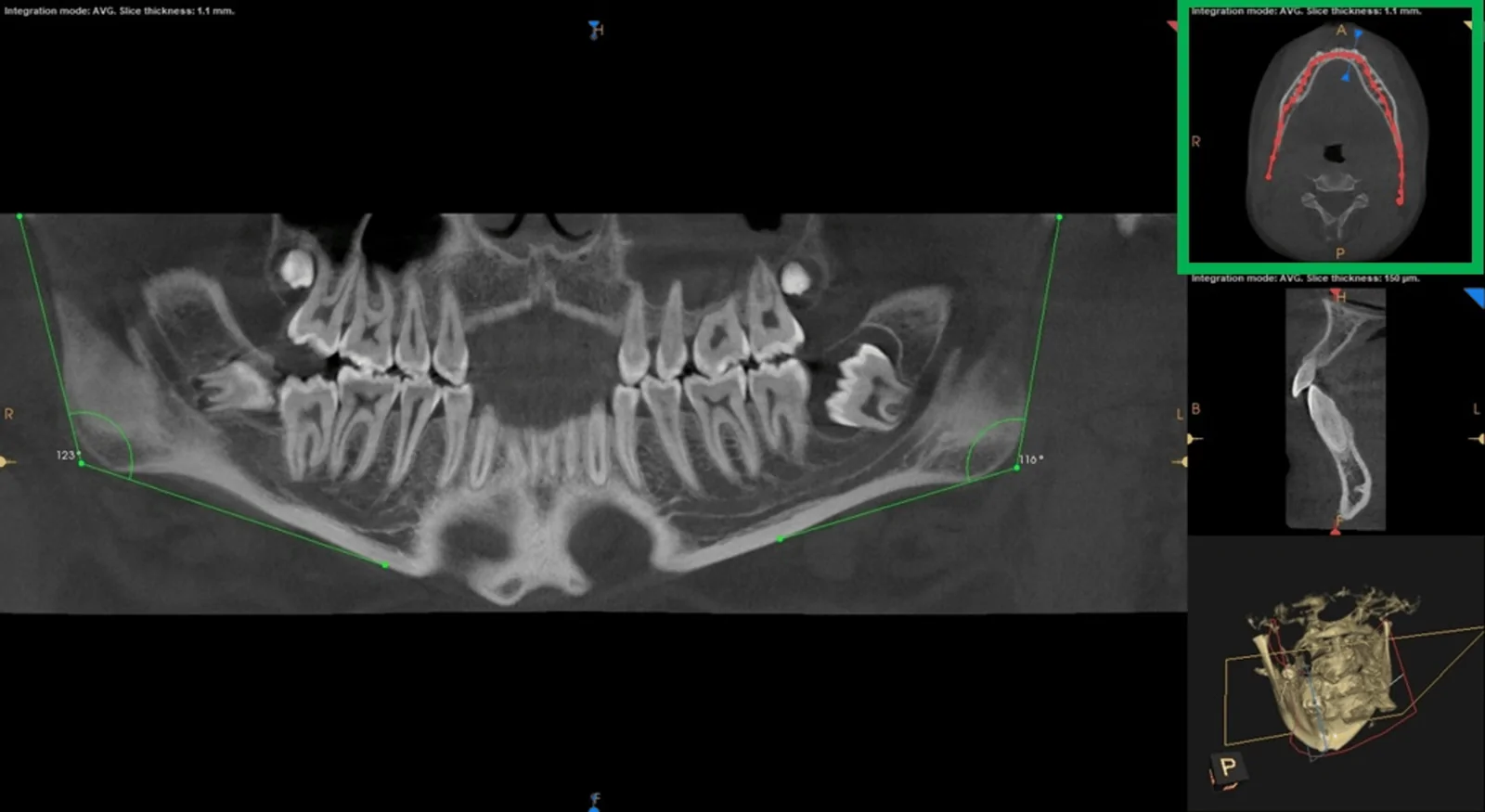
CBCT image of the mandible showing the right and left gonial angles (Ar-Go-Me; green), measured bilaterally between the Ar-Go line along the posterior border of the ramus and the Go-Me line along the inferior border of the mandible. Ar: Articulare; CBCT: Cone-beam computed tomography; Go: Gonion; Me: Menton.

The resulting slice was 1.1 μm thick and viewed at a magnification of 0.3 mm, displaying the mandible in the axial section with clear visualization of the three points described earlier (Figure 3).

Once this was completed, the software displayed the angular measurement between the three marked points in the display panel on the right side of the screen.

The process was repeated for the right and left sides of the images for all 24 participants until all measurements were obtained.

Statistical analysis

All statistical analyses were performed using IBM SPSS Statistics for Windows (version 23.0; IBM Corp., Armonk, New York, USA). As the angular measurements were quantitative data, they were summarized using the mean and SD. A p-value of less than 0.05 was considered statistically significant for all analyses. Bland-Altman analysis was used to evaluate agreement between the different methods.

Reliability assessment

Intra-examiner reliability was assessed using the intraclass correlation coefficient (ICC) based on absolute agreement, together with weighted kappa tests. In all sets, the intra- and inter-rater ICCs demonstrated excellent repeatability and reliability for both 2D and 3D measurements.

## Results

There was no statistically significant difference among the mean gonial angle measurements obtained from the LC (125.42° ± 3.45°), the right and left sides of the OPG (125.26° ± 3.54° and 125.78° ± 4.43°, respectively), and the right and left sides of the CBCT reconstructions (125.82° ± 4.18° and 125.85° ± 4.59°, respectively) (Table 1).

**Table 1:** Depiction of comparison of gonial angle measurements across all imaging techniques. *p < 0.05 indicates statistical significance.

Method	Statistic	LC	OPG, right	OPG, left	CBCT, right	CBCT, left
Mean ± SD (°)		125.42 ± 3.45	125.26 ± 3.54	125.78 ± 4.43	125.82 ± 4.18	125.85 ± 4.59
LC	p value	-	0.8125	0.7106	0.4737	0.6527
	t value	-	0.24	0.3757	0.7285	0.456
OPG, right	p value	0.8125	-	0.521	0.1416	-
	t value	0.24	-	0.6517	1.522	-
OPG, left	p value	0.7106	0.521	-	-	0.765
	t value	0.3757	0.6517	-	-	0.3025
CBCT, right	p value	0.4737	0.1416	-	-	-
	t value	0.7285	1.522	-	-	-
CBCT, left	p value	0.6527	-	0.765	-	-
	t value	0.456	-	0.3025	-	-

**Figure 4:**
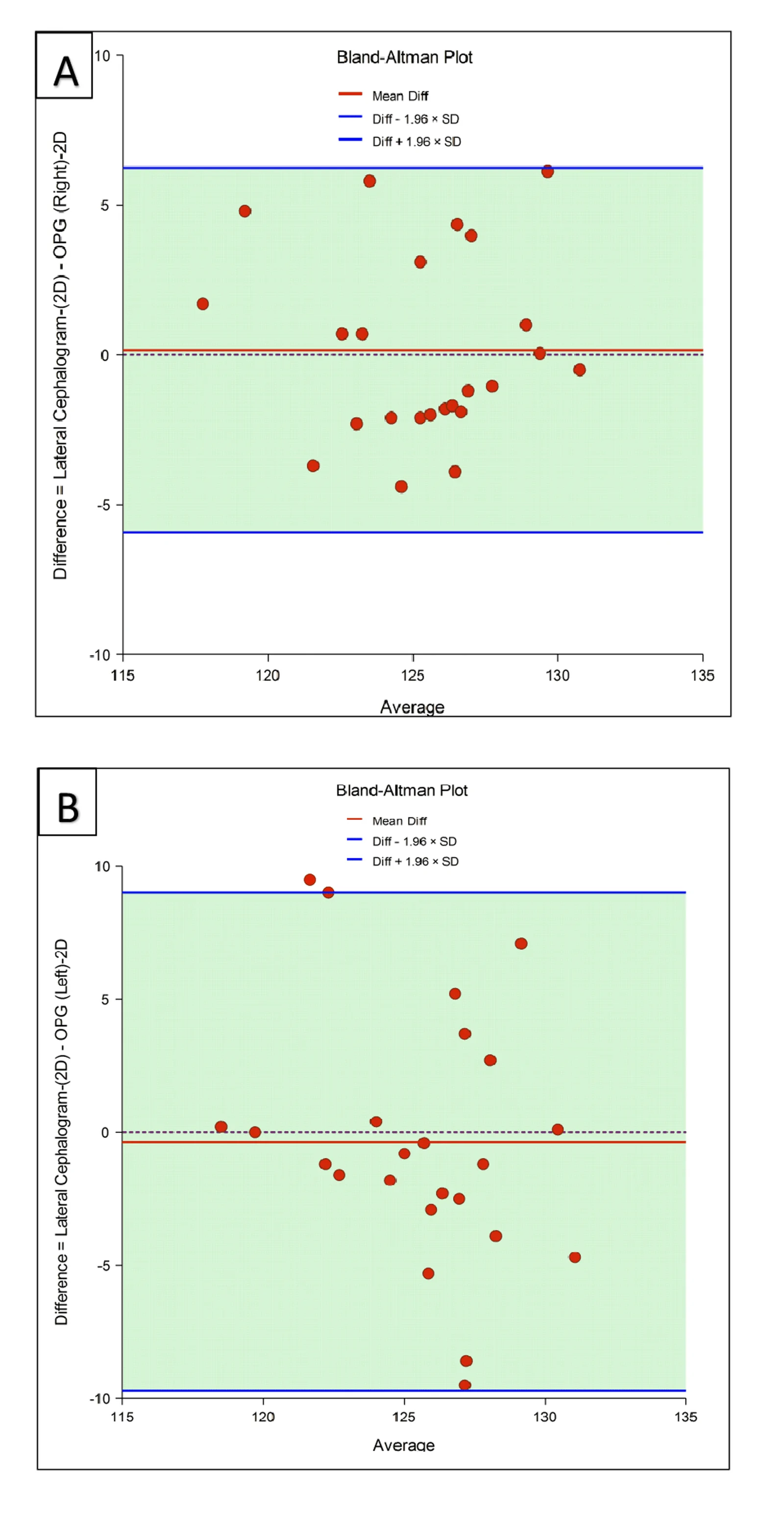
(A) Bland-Altman analysis showing the bias and limits of agreement between gonial angle measurements obtained using a 2D lateral cephalogram and right-sided measurements obtained using 3D CBCT. (B) Bland-Altman analysis showing the bias and limits of agreement between gonial angle measurements obtained using a 2D lateral cephalogram and left-sided measurements obtained using 3D CBCT. CBCT: Cone-beam computed tomography.

Bland-Altman analysis comparing the LC and right OPG measurements across 24 participants showed a near-zero mean bias of +0.15°, with the 95% confidence interval (-1.16° to +1.46°) including zero, indicating no statistically significant systematic difference between the two methods on average (Figure 4). All five assessment methods demonstrated statistically significant inter-examiner agreement (p < 0.0001), with weighted kappa values ranging from 0.8713 to 0.9741, indicating high to near-perfect agreement beyond chance. The 2D methods consistently demonstrated higher inter-examiner agreement than their 3D counterparts. The right OPG measurement showed the highest agreement (weighted kappa = 0.9741; 99.17% observed agreement), followed closely by the LC (weighted kappa = 0.9709; 99.00% observed agreement) and the left OPG measurement (weighted kappa = 0.9531; 98.46% observed agreement). The 3D CBCT measurements demonstrated comparatively lower, although still high, agreement, with the right side showing a weighted kappa of 0.9174 and 97.17% observed agreement and the left side showing the lowest agreement among all methods, with a weighted kappa of 0.8713 and 95.69% observed agreement. Notably, expected agreement due to chance remained relatively stable across all methods at approximately 65%-68%, whereas observed agreement progressively declined from the 2D to the 3D methods, with the left CBCT measurement showing the lowest observed agreement. Overall, weighted kappa analysis indicated near-perfect examiner agreement for the 2D assessments and high but comparatively lower inter-examiner consistency for the 3D assessments, particularly on the left side.

**Figure 5:**
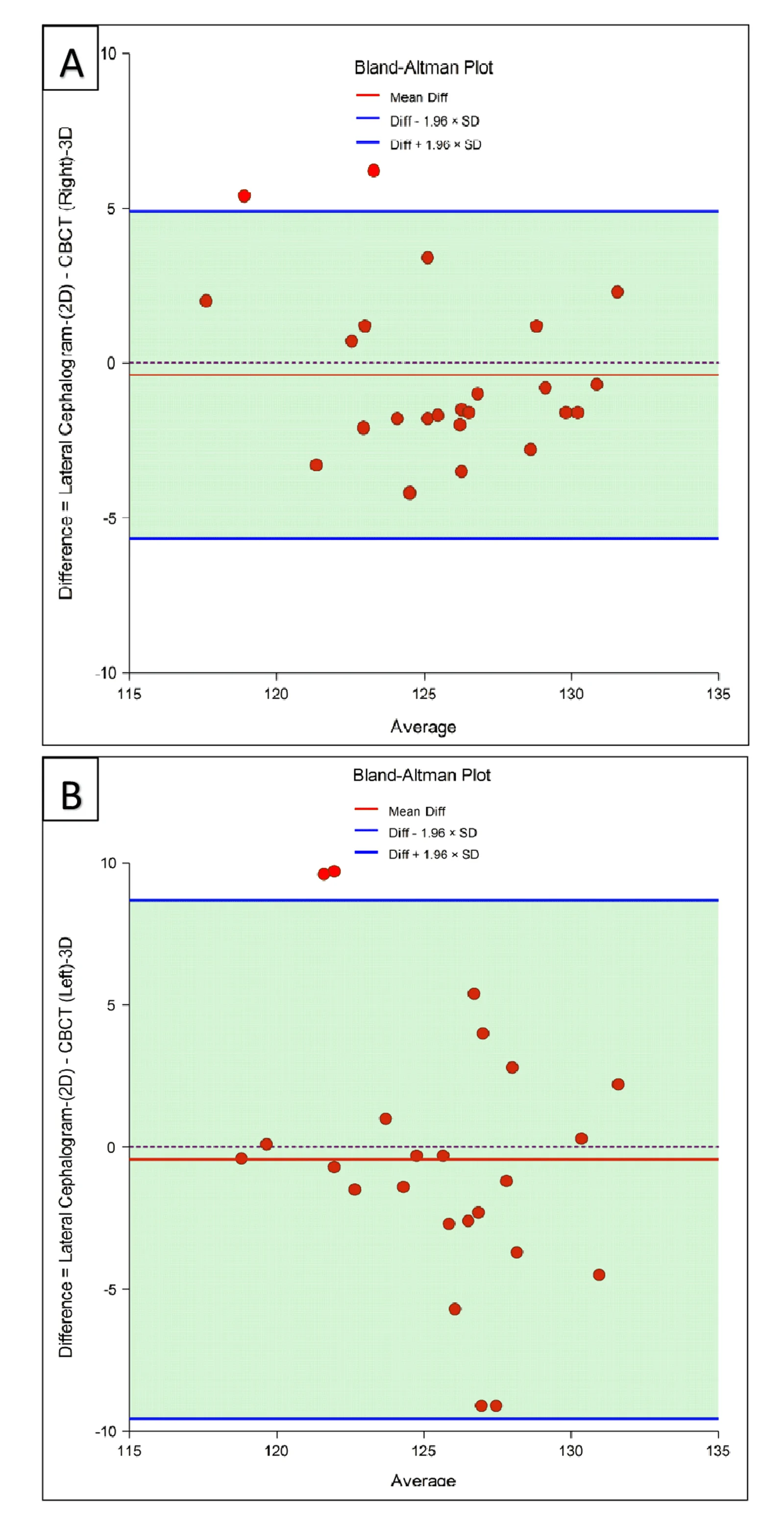
(A) Bland-Altman analysis showing the bias and limits of agreement between gonial angle measurements obtained using a 2D lateral cephalogram and right-sided measurements obtained using 3D CBCT. (B) Bland-Altman analysis showing the bias and limits of agreement between gonial angle measurements obtained using a 2D lateral cephalogram and left-sided measurements obtained using 3D CBCT. CBCT: Cone-beam computed tomography.

**Figure 6:**
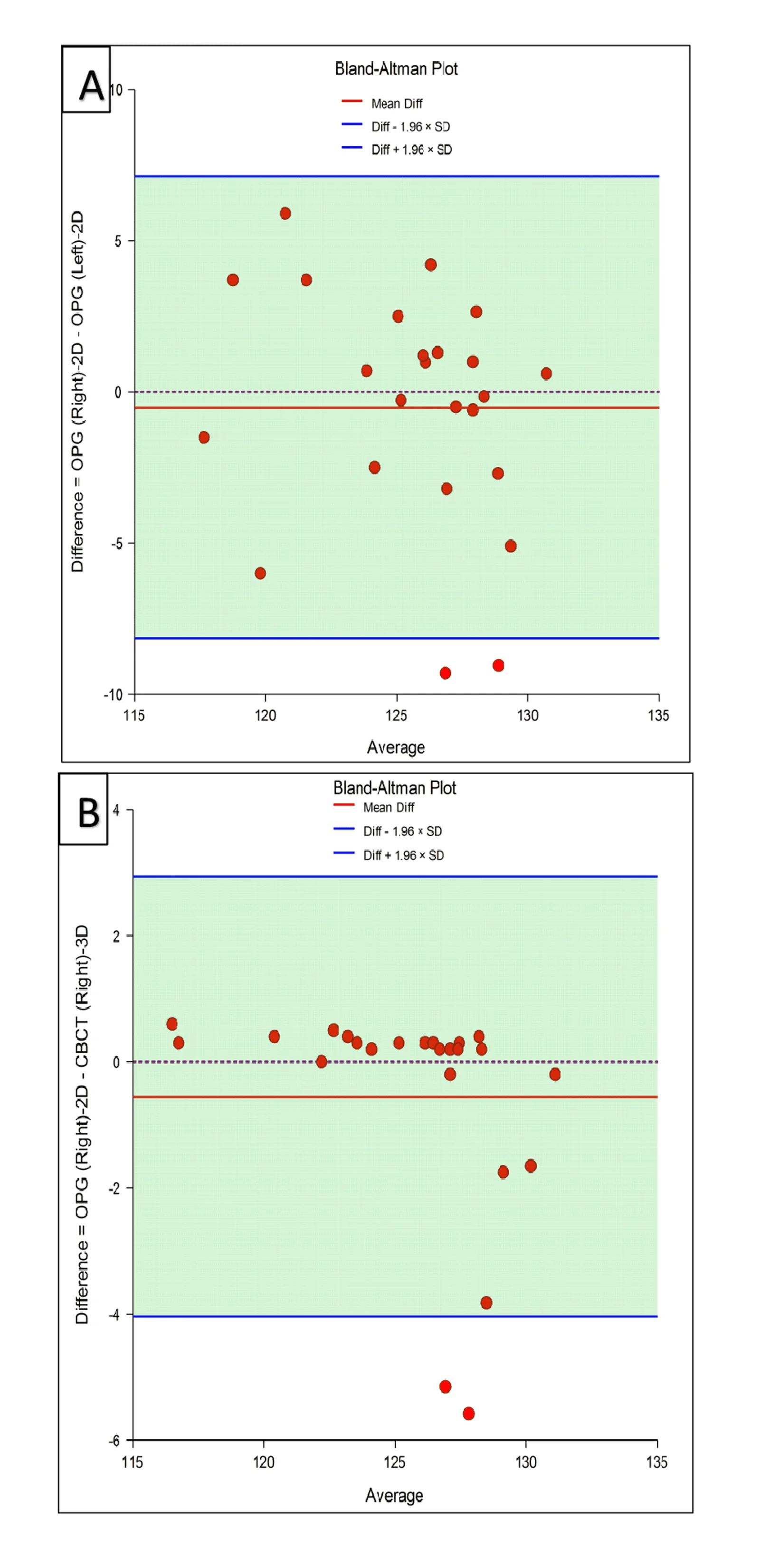
(A) Bland-Altman analysis showing the bias and limits of agreement between right- and left-sided gonial angle measurements obtained using 2D OPG. (B) Bland-Altman analysis showing the bias and limits of agreement between right-sided gonial angle measurements obtained using 2D OPG and 3D CBCT. CBCT: Cone-beam computed tomography; OPG: Orthopantomogram.

**Figure 7:**
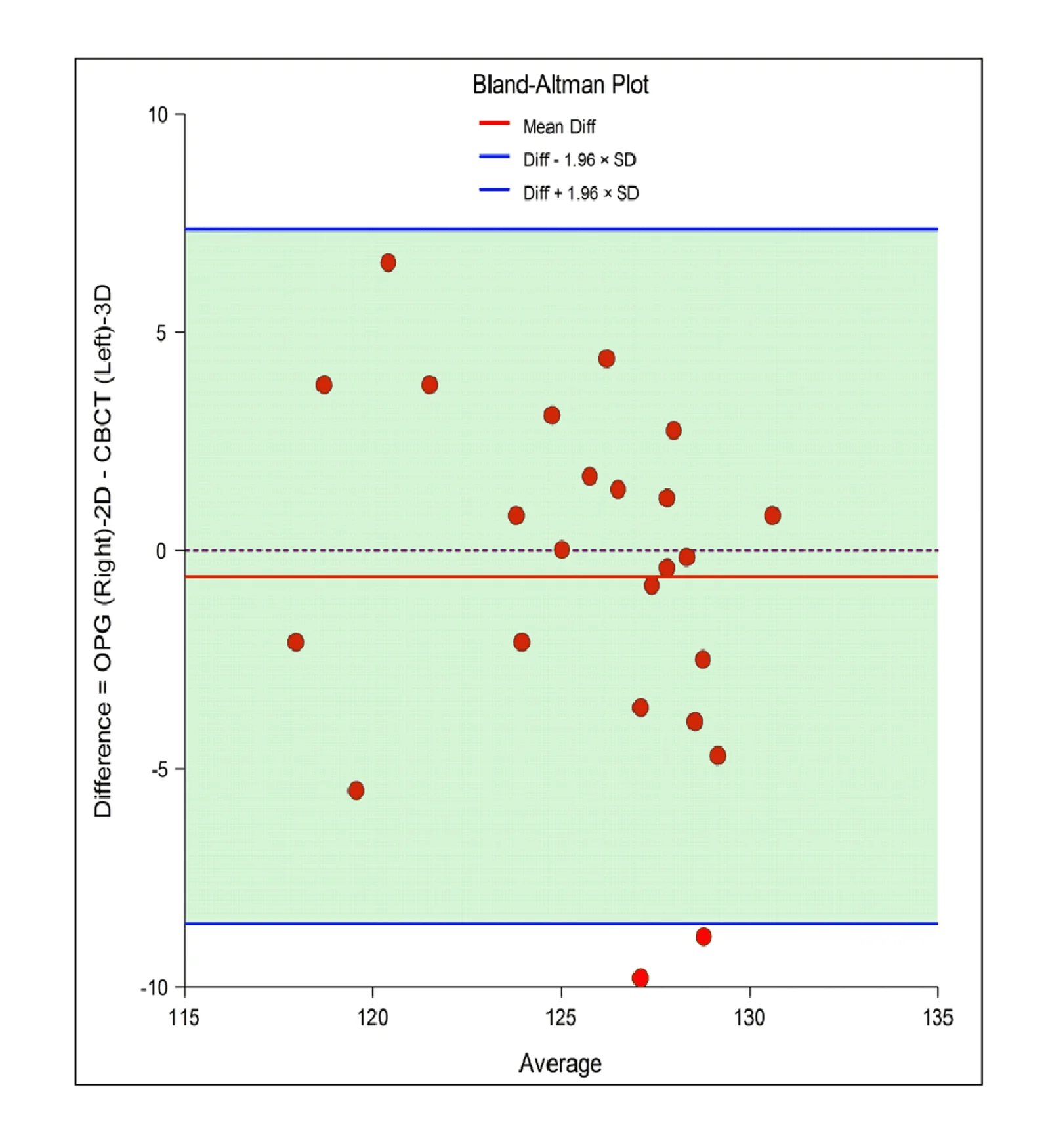
Bland-Altman analysis showing the bias and limits of agreement between right-sided gonial angle measurements obtained using 2D OPG and 3D CBCT. CBCT: Cone-beam computed tomography; OPG: Orthopantomogram.

**Figure 8:**
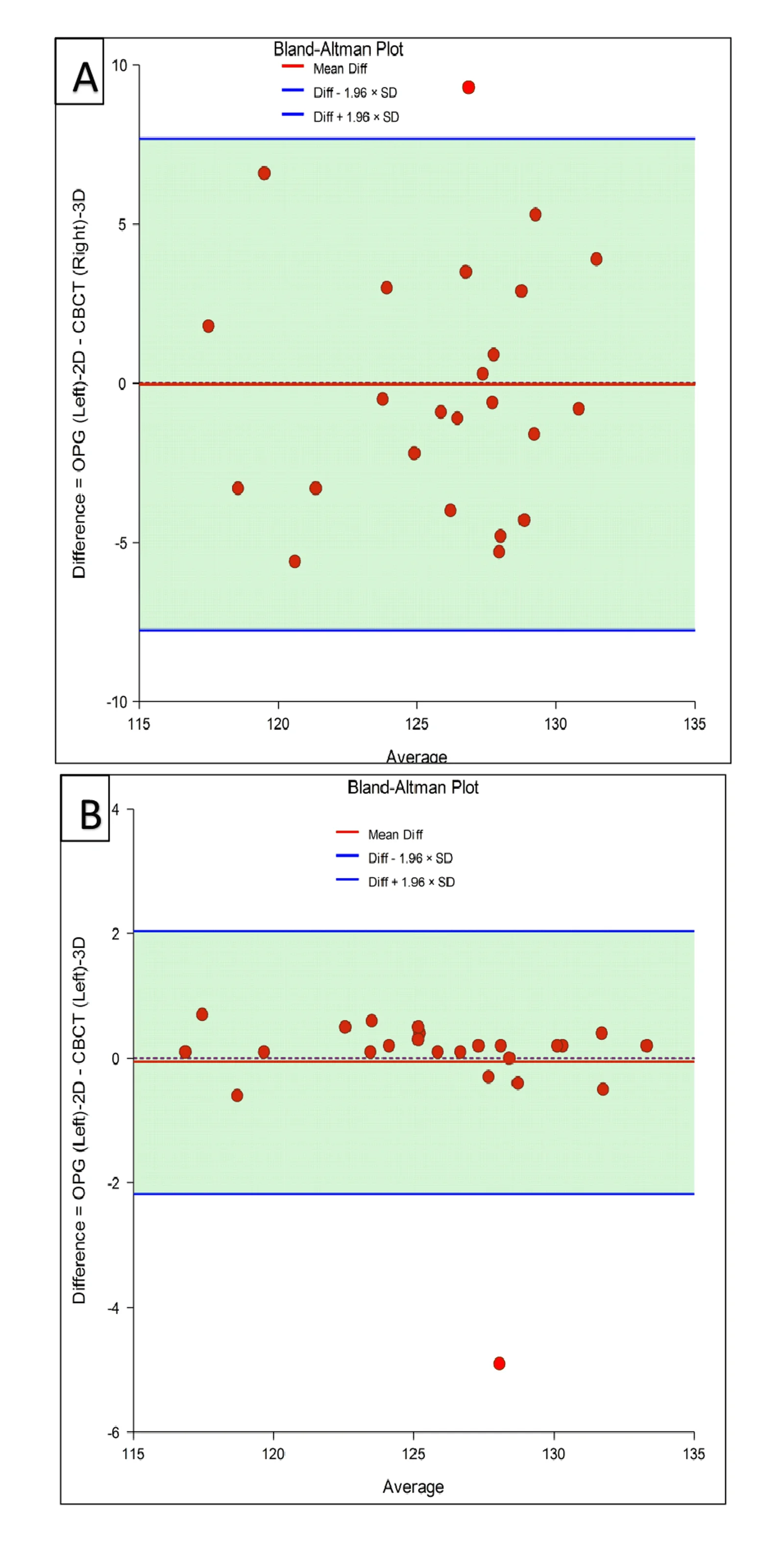
(A, B) Bland-Altman plots showing the bias and limits of agreement between left-sided gonial angle measurements obtained using 2D OPG and right-sided measurements obtained using 3D CBCT. CBCT: Cone-beam computed tomography; OPG: Orthopantomogram.

**Figure 9:**
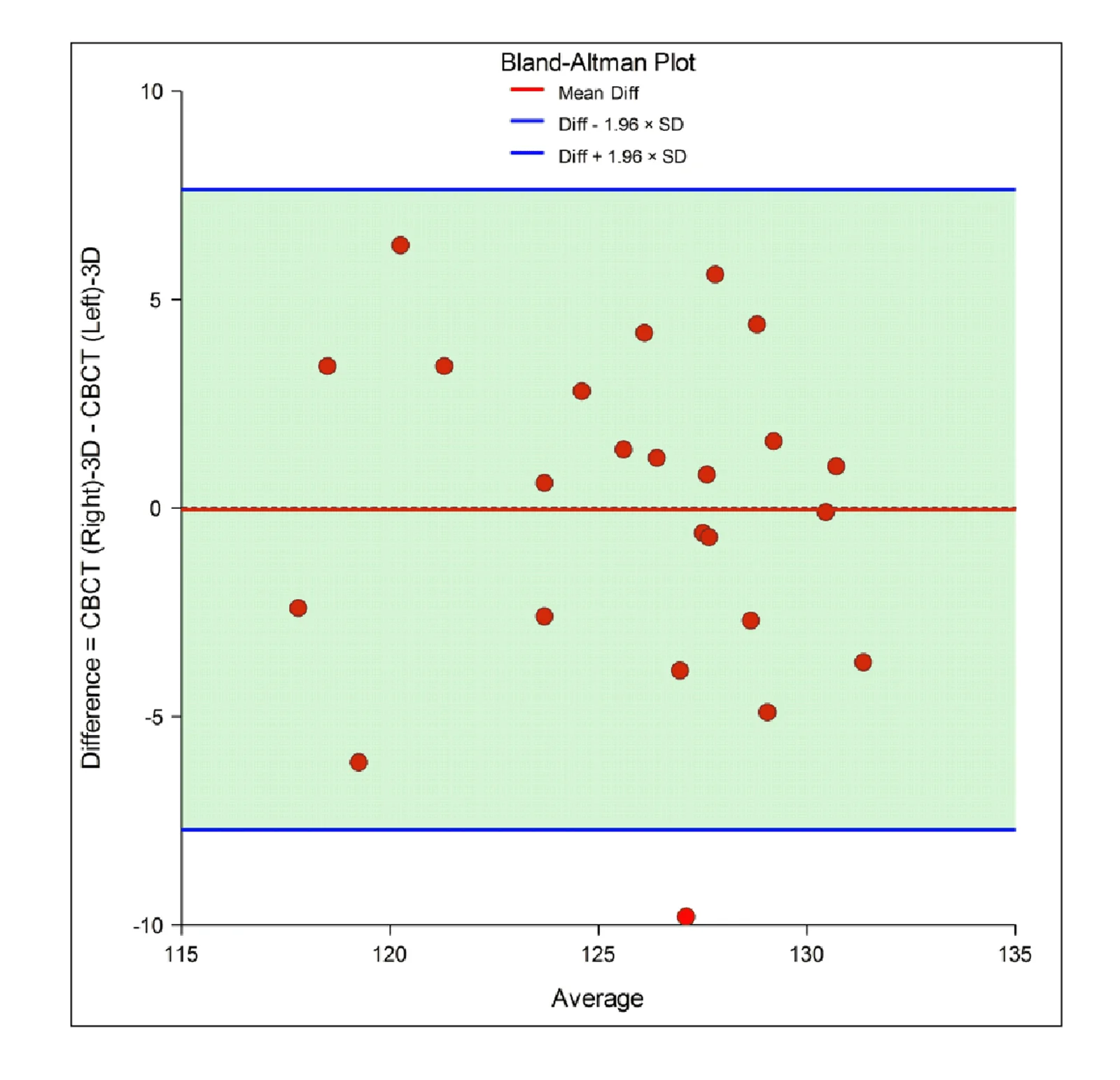
Bland-Altman analysis showing the bias and limits of agreement for right-sided gonial angle measurements obtained using 3D CBCT. CBCT: Cone-beam computed tomography.

Bland-Altman analyses conducted across other combinations of techniques showed similar findings (Figures 5-9).

All methods of gonial angle measurement demonstrated good-to-excellent reliability, although a clear difference was observed between the 2D and 3D techniques. The 2D methods, LC (ICC = 0.999), right OPG measurement (ICC = 0.997), and left OPG measurement (ICC = 0.997), demonstrated excellent reliability with narrow confidence intervals, indicating near-perfect consistency and minimal measurement error when the examiner scores were averaged. In contrast, the 3D CBCT measurements demonstrated comparatively lower reliability. The right CBCT measurement showed good-to-excellent reliability (ICC = 0.953; 95% confidence interval: 0.891-0.980), whereas the left CBCT measurement demonstrated the lowest reliability among all methods (ICC = 0.929; 95% confidence interval: 0.836-0.969), with a wider confidence interval suggesting greater inter-examiner variability. Overall, averaging the examiner scores resulted in excellent reliability for the 2D assessments, whereas the 3D assessments, particularly on the left side, showed greater measurement uncertainty despite retaining good-to-excellent reliability (Table 2).

**Table 2:** Inter-observer reliability of different assessment procedures between Examiner 1 and Examiner 2, evaluated using the intraclass correlation coefficient (ICC). CBCT: Cone-beam computed tomography; df: Degrees of freedom; ICC: Intraclass correlation coefficient; OPG: Orthopantomogram.

Method	Measure type	ICC	Lower bound	Upper bound	F value	df1	df2	p value
Lateral cephalogram (2D)	Single measures	0.998	0.995	0.999	924.158	23	23	<0.0001
Lateral cephalogram (2D)	Average measures	0.999	0.997	1	924.158	23	23	<0.0001
OPG, right side (2D)	Single measures	0.995	0.987	0.998	363.236	23	23	<0.0001
OPG, right side (2D)	Average measures	0.997	0.994	0.999	363.236	23	23	<0.0001
OPG, left side (2D)	Single measures	0.993	0.985	0.997	296.228	23	23	<0.0001
OPG, left side (2D)	Average measures	0.997	0.992	0.999	296.228	23	23	<0.0001
CBCT, right side (3D)	Single measures	0.91	0.804	0.96	21.226	23	23	<0.0001
CBCT, right side (3D)	Average measures	0.953	0.891	0.98	21.226	23	23	<0.0001
CBCT, left side (3D)	Single measures	0.867	0.718	0.94	14.08	23	23	<0.0001
CBCT, left side (3D)	Average measures	0.929	0.836	0.969	14.08	23	23	<0.0001

There was statistically significant inter-examiner agreement (p < 0.0001), with weighted kappa values ranging from 0.8713 to 0.9741, indicating substantial to near-perfect agreement beyond chance. The 2D methods consistently outperformed their 3D counterparts: the right OPG measurement showed the highest agreement (kappa = 0.9741; 99.17% observed agreement), followed closely by the LC (kappa = 0.9709; 99.00% observed agreement) and the left OPG measurement (kappa = 0.9531; 98.46% observed agreement). The 3D CBCT measurements demonstrated comparatively lower agreement, with the right side showing substantial agreement (kappa = 0.9174; 97.17% observed agreement) and the left side exhibiting the lowest agreement among all procedures (kappa = 0.8713; 95.69% observed agreement). Overall, weighted kappa analysis confirmed that the 2D assessments yielded near-perfect examiner reliability, whereas the 3D assessments, particularly on the left side, showed good but comparatively lower inter-examiner consistency, although all values remained clinically acceptable (Table 3).

**Table 3:** Inter-observer reliability of different assessment procedures between Examiner 1 and Examiner 2, evaluated using weighted kappa.

Method	Measure type	ICC	95% CI, lower bound	95% CI, upper bound	F value	df1
Lateral cephalogram (2D)	Single measures	0.964	0.919	0.984	54.982	23
Lateral cephalogram (2D)	Average measures	0.982	0.958	0.992	54.982	23
OPG, right side (2D)	Single measures	0.974	0.941	0.989	76.059	23
OPG, right side (2D)	Average measures	0.987	0.97	0.994	76.059	23

Across the conventional 2D and 3D methods of gonial angle estimation, excellent intra-observer reliability was observed (ICC > 0.98), with statistically significant agreement (p < 0.0001) between the test and retest sessions when the examiner scores were averaged. The 2D methods demonstrated uniformly high reliability, with the left OPG measurement achieving near-perfect consistency (ICC = 0.999; 95% CI: 0.997-0.999), followed by the LC (ICC = 0.982; 95% CI: 0.958-0.992) and the right OPG measurement (ICC = 0.987; 95% CI: 0.970-0.994). Interestingly, unlike the inter-observer results, in which the 2D methods outperformed the 3D methods, the 3D CBCT measurements demonstrated equally excellent or, in some cases, superior intra-observer reliability. The right CBCT measurement (ICC = 0.999; 95% CI: 0.997-0.999) and the left CBCT measurement (ICC = 0.998; 95% CI: 0.996-0.999) both achieved near-perfect consistency, comparable to the best 2D measurements. The CIs for all methods were consistently narrow, with the lower bounds never falling below 0.958, indicating high precision in the reliability estimates. Overall, averaging the test and retest scores resulted in excellent within-examiner consistency across all procedures, with the 3D CBCT methods performing equally well as the 2D methods in this intra-observer context, in contrast to the inter-observer findings, in which the 3D methods showed greater variability between different examiners (Table 4).

**Table 4:** Intra-observer reliability of different assessment procedures between test and retest measurements, evaluated using the ICC. CBCT: Cone-beam computed tomography; df: Degrees of freedom; ICC: Intraclass correlation coefficient; OPG: Orthopantomogram.

Method	Measure type	ICC	Lower bound	Upper bound	F value	df1	df2	p value
Lateral cephalogram (2D)	Single measures	0.964	0.919	0.984	54.982	23	23	<0.0001
Lateral cephalogram (2D)	Average measures	0.982	0.958	0.992	54.982	23	23	<0.0001
OPG, right side (2D)	Single measures	0.974	0.941	0.989	76.059	23	23	<0.0001
OPG, right side (2D)	Average measures	0.987	0.97	0.994	76.059	23	23	<0.0001
OPG, left side (2D)	Single measures	0.997	0.994	0.999	734.315	23	23	<0.0001
OPG, left side (2D)	Average measures	0.999	0.997	0.999	734.315	23	23	<0.0001
CBCT, right side (3D)	Single measures	0.998	0.994	0.999	809.027	23	23	<0.0001
CBCT, right side (3D)	Average measures	0.999	0.997	0.999	809.027	23	23	<0.0001
CBCT, left side (3D)	Single measures	0.997	0.993	0.999	644.808	23	23	<0.0001
CBCT, left side (3D)	Average measures	0.998	0.996	0.999	644.808	23	23	<0.0001

All five methods demonstrated statistically significant intra-examiner agreement (p < 0.0001), with weighted kappa values ranging from 0.8466 to 0.9616, indicating substantial to near-perfect consistency between the test and retest sessions. The 3D CBCT methods showed the highest agreement, with the right CBCT measurement demonstrating the strongest reliability (kappa = 0.9616; 98.67% observed agreement), followed closely by the left CBCT measurement (kappa = 0.9526; 98.33% observed agreement) and the left OPG measurement (kappa = 0.9505; 98.33% observed agreement). The LC showed good agreement (kappa = 0.8734; 95.83% observed agreement), whereas the right OPG measurement demonstrated the lowest reliability among all methods (kappa = 0.8466; 94.44% observed agreement), although it remained within the substantial agreement range. Expected agreement due to chance remained relatively consistent across all methods at approximately 64%-67%, with observed agreement being considerably higher in all cases. Notably, unlike the inter-examiner weighted kappa findings, in which the 2D methods outperformed the 3D methods, the intra-examiner results showed that the 3D CBCT methods achieved the highest or comparable reliability. This finding suggests that individual examiners produced highly consistent repeated measurements when using 3D imaging, whereas variability between different examiners was more pronounced for the 3D assessments. Overall, all methods demonstrated clinically acceptable intra-examiner reliability, with the right CBCT measurement being the most consistent and the right OPG measurement being the least consistent, although the latter still demonstrated substantial agreement (Table 5).

**Table 5:** Intra-observer reliability of different assessment procedures between test and retest measurements, evaluated using weighted kappa. CBCT: Cone-beam computed tomography; OPG: Orthopantomogram.

Method	Observed agreement	Expected agreement by chance	Weighted kappa	Standard error	z value	p value
Lateral cephalogram (2D)	95.83%	67.10%	0.8734	0.1269	6.88	<0.0001
OPG, right side (2D)	94.44%	63.77%	0.8466	0.1326	6.38	<0.0001
OPG, left side (2D)	98.33%	66.33%	0.9505	0.1271	7.48	<0.0001
CBCT, right side (3D)	98.67%	65.44%	0.9616	0.1281	7.51	<0.0001
CBCT, left side (3D)	98.33%	64.86%	0.9526	0.1296	7.35	<0.0001

## Discussion

The present study evaluated the agreement between gonial angle measurements obtained from conventional LCs, OPGs, and CBCT-reconstructed cephalograms and panoramic radiographs in normodivergent individuals. The principal finding was that there were no statistically significant differences among the measurements obtained using the four imaging methods. Furthermore, Bland-Altman analysis demonstrated minimal systematic bias with acceptable limits of agreement, indicating that CBCT-derived reconstructed images provide measurements comparable to those obtained from conventional radiographs. These findings are consistent with those of Jadhav A et al. [20], suggesting that CBCT-generated 2D reconstructions may serve as a reliable alternative to conventional cephalometric and panoramic radiographs when a CBCT scan has already been acquired for clinical purposes. Interestingly, Baldini B et al. [6] demonstrated that cephalometric measurements obtained directly from CBCT are highly reliable and comparable to those obtained from reconstructed LCs when measurements are derived from midline landmarks of the skull, such as nasion (N), sella (S), A point, B point, anterior nasal spine (ANS), posterior nasal spine (PNS), and menton. These landmarks are not affected by left-right superimposition. However, they found that measurements involving bilateral structures, such as gonion-menton (Go-Me) and gonion-sella (Go-S), were more affected and therefore had lower accuracy, which directly contrasts with the results obtained in our study.

The gonial angle is an important cephalometric parameter used in orthodontic diagnosis because it reflects mandibular morphology, facial growth pattern, and mandibular rotation [1]. Accurate assessment of this angle contributes to diagnosis, treatment planning [2], and forensic identification [3]. Conventional LCs have traditionally been used to evaluate the gonial angle [6]; however, the superimposition of bilateral structures and geometric distortion may reduce measurement accuracy [7]. Panoramic radiographs partially overcome this limitation by allowing separate visualization of the right and left gonial angles [8-11], whereas CBCT provides true 3D visualization of the craniofacial skeleton without superimposition [13].

The absence of statistically significant differences between conventional and CBCT-derived measurements observed in this study supports the growing evidence that reconstructed 2D images generated from CBCT volumes preserve cephalometric accuracy. Similar observations have been reported by Nalçaci R et al., Bholsithi W et al., Chang ZC et al., Damstra J et al., Zamora N et al., and Yitschaky O et al. [21-26], who demonstrated that angular measurements obtained from CBCT-derived cephalograms are comparable to those obtained from conventional cephalograms. Likewise, Kumar V et al. [27] reported excellent agreement between conventional and CBCT-synthesized cephalograms, concluding that CBCT reconstructions can reliably reproduce routine cephalometric measurements. Collectively, these studies suggest that modern CBCT reconstruction algorithms maintain anatomical fidelity while minimizing projection errors associated with conventional imaging.

An important strength of the present study is the use of Bland-Altman analysis in addition to conventional hypothesis testing. While paired statistical tests determine whether significant differences exist between mean measurements, Bland-Altman analysis evaluates the degree of agreement between techniques and therefore provides a more clinically meaningful assessment of interchangeability. The negligible mean bias and narrow limits of agreement observed in the present investigation indicate that the small differences between imaging methods are unlikely to influence clinical decision-making. This finding reinforces the practical equivalence of conventional radiographs and CBCT-derived reconstructed images for gonial angle assessment.

The reliability analysis further supports the clinical applicability of the findings. Excellent intra- and inter-examiner agreement was observed across all imaging methods. Although conventional 2D radiographs demonstrated marginally higher inter-examiner agreement than CBCT reconstructions, CBCT exhibited equally high and, in some analyses, superior intra-examiner reliability. This suggests that once examiners become familiar with landmark identification on reconstructed CBCT images, measurements can be reproduced consistently. The slightly lower inter-examiner agreement for CBCT may reflect the learning curve associated with 3D image manipulation rather than limitations of the imaging modality itself.

From a clinical perspective, these findings have important implications. CBCT examinations are increasingly prescribed in orthodontics for impacted teeth, temporary anchorage device planning, craniofacial anomalies, airway assessment, and orthognathic surgical planning [28]. When such scans are already available, the present findings suggest that reconstructed cephalograms and panoramic images can be used for gonial angle assessment without requiring additional conventional radiographs. This approach is consistent with the “as low as reasonably achievable” (ALARA) and “as low as diagnostically acceptable, being indication-oriented and patient-specific” (ALADAIP) principles by avoiding unnecessary radiation exposure while maximizing the diagnostic information obtained from a single imaging examination.

The present study has several methodological strengths. All radiographs were acquired using the same imaging system under standardized exposure parameters, minimizing equipment-related variability. Measurements were performed using dedicated Carestream imaging software under standardized protocols, and examiner reliability was evaluated comprehensively using intraclass correlation coefficients, weighted kappa statistics, and Bland-Altman analysis. Together, these measures strengthen the internal validity of the study and increase confidence in the reproducibility of the reported findings.

Nevertheless, several limitations should be acknowledged. The sample size was relatively small and consisted exclusively of normodivergent skeletal Class I individuals. Consequently, the findings cannot be directly generalized to individuals with hyperdivergent or hypodivergent growth patterns, skeletal Class II or Class III malocclusions, facial asymmetry, or craniofacial anomalies. Furthermore, only the gonial angle was evaluated. Additional cephalometric variables involving complex craniofacial relationships may demonstrate different levels of agreement and warrant independent investigation. Future studies involving larger and more diverse populations, multicenter datasets, and comprehensive cephalometric analyses would provide stronger evidence regarding the interchangeability of conventional and CBCT-derived imaging.

Overall, the findings of this study indicate that CBCT-reconstructed cephalograms and panoramic radiographs provide gonial angle measurements that closely agree with those obtained from conventional radiographs while demonstrating high reproducibility. These results support the selective use of CBCT-derived reconstructed images in orthodontic diagnosis when CBCT data are already available, thereby reducing additional radiation exposure without compromising measurement accuracy.

## Conclusions

Within the limitations of this cross-sectional study, gonial angle measurements obtained from CBCT-reconstructed cephalograms and panoramic images demonstrated excellent agreement with those obtained from conventional LCs and OPGs in normodivergent individuals. No statistically significant differences were observed between the imaging modalities, indicating that CBCT-derived reconstructed images provide comparable measurements for gonial angle assessment.

These findings suggest that when a CBCT scan has already been acquired for appropriate clinical indications, reconstructed cephalometric and panoramic images may be used reliably for gonial angle evaluation, thereby avoiding the need for additional conventional radiographs and reducing unnecessary radiation exposure in accordance with radiation protection principles.

Further studies involving larger sample sizes, diverse skeletal patterns, and additional cephalometric variables are warranted to establish the broader applicability of CBCT-reconstructed images in routine orthodontic diagnosis and treatment planning.
